# A bibliometric analysis of insomnia in adolescent

**DOI:** 10.3389/fpsyt.2023.1246808

**Published:** 2023-10-25

**Authors:** Tianci Gao, Yulei Tao, Qianfei Wang, Jiayi Liu, Zekun Du, YueYi Xing, Fenqiao Chen, Jianqiang Mei

**Affiliations:** ^1^Graduate School, Hebei University of Chinese Medicine, Shijiazhuang, Hebei, China; ^2^First Affiliated Hospital, Hebei University of Chinese Medicine, Shijiazhuang, Hebei, China; ^3^School of Basic Medicine, Hebei University of Chinese Medicine, Shijiazhuang, Hebei, China

**Keywords:** bibliometrics, insomnia, adolescent, CiteSpace, VOSViewer

## Abstract

**Background:**

The negative effects of insomnia on adolescents’ development, academic performance, and quality of life place a burden on families, schools, and society. As one of the most important research directions for insomnia, adolescent insomnia has significant research value, social value, and practical significance. Unfortunately, there is no bibliometric analysis in this field of study. This study aims to analyze published articles using bibliometrics, summarize the current research progress and hot topics in this field systematically and exhaustively, and predict the future direction and trend of research.

**Methods:**

For this study, the Web of Science Core Collection (WoSCC) database was searched between 2002 and 2022 for publications related to adolescent insomnia. The R–bibliometrix, VOSViewer, and CiteSpace software were utilized for bibliometric analysis.

**Results:**

This investigation included 2468 publications from 3102 institutions in 87 countries, led by China and the United States. This field of research has entered a period of rapid development since 2017. The journal with the most publications on adolescent insomnia is *Sleep*, which is also the most co–cited journal. *American Journal of Psychology* has the highest impact factor among the top 10 journals. These papers were written by 10605 authors; notably, Liu Xianchen emerged as the author with the highest frequency of publications, while Mary A. Carskadon was the most frequently co–cited author. Mental health and comorbid diseases were the main research directions in this field. “Depression,” “anxiety,” “mental health,” “COVID–19,” “stress,” “quality of life,” “heart rate variability,” and “attention–deficit hyperactivity disorder” were hot spots and trends in this field at the current moment.

**Conclusion:**

The research on adolescent insomnia has social value, research value, and research potential; its development is accelerating, and an increasing number of researchers are focusing on it. This study summarized and analyzed the development process, hot spots, and trends of adolescent insomnia research using bibliometric analysis, which identified the current hot topics in this field and predicted the development trend for the future.

## Introduction

Insomnia is a condition characterized by dissatisfaction with the quantity or quality of sleep and has the following three main symptoms: (1) challenges falling asleep, with an inability to fall asleep within 20–30 min; (2) inability to maintain sleep, with frequent waking during sleep (for more than 20–30 min) and difficulty returning to sleep after waking; (3) early–morning wakefulness, with waking at least 30 min before the desired time and before sleep reaches 6.5 hours (1). According to several studies, the prevalence of insomnia is about 23–40%, and it is more common in women than men (2–4). Adolescents are defined as people 10–24 years of age, which is more consistent with the common definitions of adolescent development and adolescence as well as our understanding of adolescence than the range of 19–24 that is sometimes used (5). Adolescent insomnia is a sleep disorder that affects young people, typically teenagers (6). The prevalence of insomnia in adolescents has been shown to be about 24% (16–19 years of age; Diagnostic and Statistical Manual of Mental Disorders, 4th Edition [DSM–IV]) (7) and 25% (19–24 years of age; criteria from DSM–IV) (8); similarly, insomnia is more common in women than in men (8, 9). Insomnia can also lead to comorbidity with mental disorders such as depression and increase the severity of depressive symptoms (10). There are many risk factors for insomnia in adolescents, such as sex (11), puberty (12, 13), use of electronic devices (14), stress (15), social environmental factors (16), and mental health (17). Insomnia can cause many adverse consequences and induce conditions such as obesity (18), depression (19), suicidal ideation (20), cardiometabolic disease (21), and declines in cognitive development and learning abilities (22). Insomnia has a significant influence on teenagers, and many adolescents experience it. There are two main approaches to treating insomnia in adolescents: cognitive behavioral interventions or medicine alone and the combination of these two therapies, and research on the effects of treatment in this population has mostly been absent (6). In a survey of 290 teenagers, 40% reported insomnia; nevertheless, only approximately 22% of adolescents seek treatment for sleep problems; 2% have used prescription or over–the–counter medications, psychological and behavioral treatments have never been applied (23). To summarize, adolescent insomnia significant influences academic performance, life, and growth, with the effects on growth mainly manifested in physical and mental health (24, 25). The following conditions and symptoms can be caused by adolescent insomnia: eating disorders (26), obesity (27), anxiety/depression, attention problems and aggressive behavior (28), alcohol and substance use disorders (29), and risk–taking behavior such as violence or suicidal ideation (30). As a result, this is a topic worth investigating, even though adolescents, parents, and society continue to pay insufficient attention to adolescent insomnia.

Recent years have seen a gradual increase in the study of adolescent insomnia, indicating that scholars have begun to pay more attention to this field; consequently, a great deal of research has been conducted on this subject. Unfortunately, the growing body of research literature makes it difficult for researchers to capture the conclusions of current research and future trends quickly. Therefore, innovative approaches are required to structure the knowledge from published research (31). Bibliometrics is an innovative approach (analyzing research literature production) to this, in addition to bibliometric mapping, which is used in bibliometric analysis and defined as a quantitative investigation of bibliographic features of document subjects to discover patterns of scientific document production in certain disciplines. It is used to visualize structures and patterns in the development of research literature (32). This study used bibliometric mapping to generate meaningful and more digestible information on adolescent insomnia research in the following ways: 1) its application in medicine enables people to analyze an enormous number of publications and their production models at both the macro and micro levels (33); 2) it can use the combination of qualitative and quantitative analysis to analyze the clusters and identify themes, legal development, knowledge base, research status, research trends, and research hot spots (34). We have commonly used bibliometric software such as CiteSpace, VOSViewer, and R–bibliometrix to cluster and analyze detailed information such as annual publication numbers, countries, institutions, journals, authors, references, keywords, hot spots, and topic trends in certain research fields. To the best of our knowledge, bibliometric studies have been conducted on cancer insomnia (35), insomnia in older adults (36), cognitive behavioral therapy for insomnia (37), and global trends in insomnia (38). However, bibliometric studies on adolescent insomnia have not yet been published. We performed a bibliometric analysis of publications on adolescent insomnia to identify and analyze the following: historical changes in literature output, subjects of research (countries and institutions), and significant authors, journals, and publications. To generate new knowledge about adolescent insomnia research, such as identifying trends in publications over time; major contributing countries and organizations; most influential authors and literature; past research targets; current research status, priorities, and trends, and predictions of future research hot spots. Further, this provides a bibliometric report for scholars, physicians, and medical students interested in adolescent insomnia.

The purpose of this study was to conduct a bibliometric analysis of publications on adolescent insomnia over the past 21 years (2002–2022) to identify the countries, institutions, journals, references and authors who have made significant contributions to the field and to predict research trends and future developments.

## Materials and methods

### Search strategy and data cleaning

**FIGURE 1:**
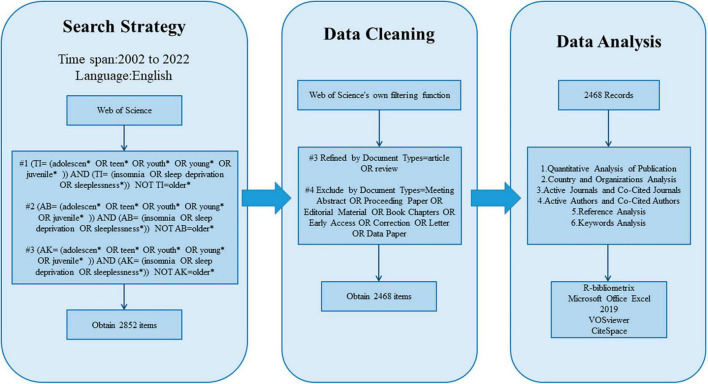
Data retrieval flow chart.

The data used in this study were collected from the Web of Science Core Collection (WoSCC) database from 1 January 2002 to 31 December 2022. The search formula is shown in Figure 1. There were a total of 2852 documents detected; the Web of Science’s filtering capabilities were used to refine items by document type (article/review) and exclude certain document types (conference abstract/conference paper/editorial material/book chapter/Early Access/correction/letter/data paper); in total, 384 items were removed, and 2468 items were included.

### Data extraction

All data collection was conducted on 19 April 2023 through searching the WoSCC. All content was recorded, including titles, authors, sources, abstracts, addresses, affiliations, document types, keywords, cited references, and years of publication. For further analysis, the chosen record contents were exported as “full record and cited references.” As CiteSpace and VOSViewer can only approve *.txt versions, and the R–bibliometrix is compatible with *.txt versions, these text files were renamed to “Download *.txt.”

### Data analysis

The quartiles and impact factors for the publications in this study were derived from Journal Citation Reports 2021.

In this study, we imported the txt files into CiteSpace, VOSViewer and R–bibliometrix for automated analysis by each software.

R–bibliometrix (39) (version 4.1),^1^ an open–source tool for quantitative scientometrics and bibliometrics research, was used to generate the following performance metrics: total number of documents, types of documents, publications by countries and institutions, total number of references and co–cited references, total number of keywords, and total number of authors and co–cited authors. The top 10 most productive countries, organizations, journals, and authors (with productivity defined as the total number of publications); the top 10 most influential journals, authors, and references (with influence defined as the number of co–citations of their publications); and the top 20 high–frequency keywords were then computed. R–bibliometrix was used to perform the following two steps.1) A global distribution network for publications was mapped, with the thickness of the line in the figure representing the intensity of cooperation between countries (more cooperation, thicker lines) and the intensity of the cooperation determined according to the number of times that authors from different countries appeared together on a publication. Further, 2) an analysis of topic trends and their development was computed, with topic trends representing hot spots and trends determined using frequencies in each time period.

Microsoft Office Excel (v2019; Microsoft Corp., Redmond, WA, USA) was used for quantitative analysis of published data (calculating the percentage of the total number of publications in each year and creating visualizations).

VOSViewer (40) (version 1.6.19) is a bibliometric analysis software used for bibliometric mapping and text mining. VOSViewer recognizes scientific information(such as countries and organizations, authors and co–cited authors, journals and co–cited journals, references, and keywords) in publications using text mining and then performs Visualization of Similarities (VoS) mapping, which is based on information co–occurrence analysis, to create bibliometric maps or landscapes (41). We imported text file data into VOSViewer to create a visual bibliometric network. A node in a VOSViewer map indicates an object such as a nation, institution, journal, author, or keyword. The quantity and categorization of these objects are indicated by the size and color of the node, respectively. The thickness of the lines connecting nodes shows the degree of collaborations or co–citations of the items. Nodes or lines of the same color in the different visual networks represent higher correlations, such as co–cited journals, co–cited authors, co–cited references, and keywords. The node color in the visual network diagram with the time scale represents the concentration of publications in the corresponding time period for metrics, such as countries, organizations, journals, and authors.

CiteSpace (42) (version 6.2.R3) is another bibliometric analysis and visualization software created by Professor Chen C that was used herein to create dual–map overlays and to analyze the strong citation bursts of references. The following parameters were set in CiteSpace: time slicing (from January 2002 to December 2022; years per slice = 1), node type (reference), selection criteria (g–index, *k* = 25), and pruning (pathfinder); other parameters were set to default. Dual–map overlays represented the main distribution fields of a journal and co–cited journals and the relationship between the fields; the color represented different areas; and the thickness of the lines represented the co–occurrence intensity. Strong citation bursts of references show the influence of a single citation in a certain period of time. Default parameters were used in CiteSpace to obtain references and generate visual charts; every bar indicates a year, with a red bar indicating the year of strong burstiness, strength representing the strength of influence, and endurance strength representing the most influential publications based on burst duration.

## Results

### Quantitative analysis of publications

The search strategy revealed a total of 2468 papers, including 2238 “articles” and 230 “reviews” about insomnia in adolescents over 21 years. The entire time can be divided into two periods based on the annual growth rate of the number of publications: an initial period I (2002–2016) and the period II (2017–2022). Following Supplementary Figure 1, the number of documents published in period I was rather small, with an average of roughly 73 papers published each year, but it increased each year, indicating that research on adolescent insomnia is in its early stages. In period II, the number of publications began to increase considerably, with an annual average of about 230 papers. The length of period I was 2.5 times that of period II, but period II contained more papers than period I. The number of publications in period II increased each year, making period II a period of rapid development, with a peak number of publications on adolescent insomnia of 322 in 2022. This demonstrated that the academic community has devoted increasing attention in recent years to the study of adolescent insomnia, showing its relevance.

### Country and organization analysis

**FIGURE 2:**
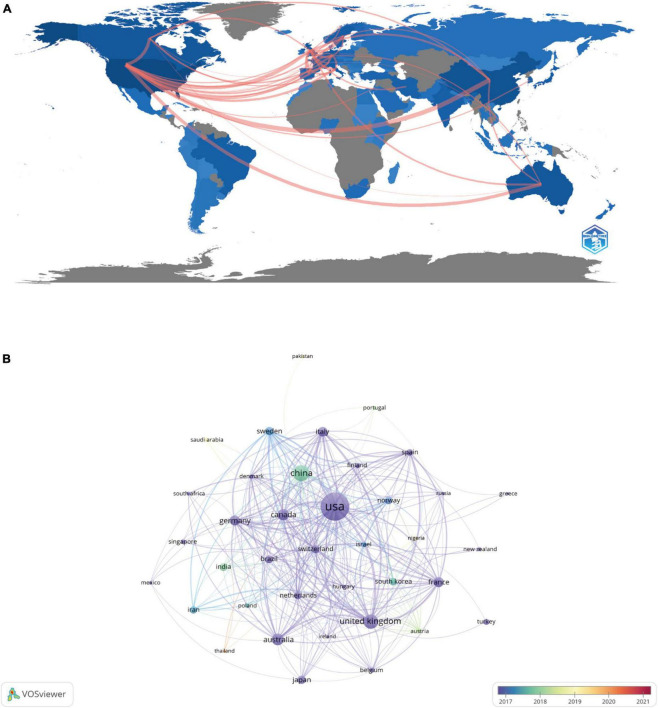
The global distribution **(A)** and intercountry visualization **(B)** of adolescent insomnia research.

The publications came from 87 countries and 3102 organizations. The top 10 countries were in North America, Asia, Europe, and Oceania, with Europe (*n* = 5), North America (*n* = 2), and Asia (*n* = 2) accounting for the majority of them (Table 1). The country with the largest number of publications among these countries was the United States (*n* = 898, 36.39%), followed by China (*n* = 303, 12.28%) and the United Kingdom (*n* = 235, 9.52%). These three countries accounted for more than half (58.58%) of all publications and were the main countries producing publications on adolescent insomnia. Following this, we filtered and visualized the 87 nations (number of publications ≥ 10) and designed a collaborative network based on each country’s number of publications and interrelationships (Figure 2). It is worth emphasizing that there was a great deal of constructive collaboration among various countries. For example, the United States collaborates closely with China, Canada, the United Kingdom, Australia, and Japan; China collaborates with the United States, Canada, the United Kingdom, Australia, and Japan; and the United Kingdom collaborates actively with the United States, France, Sweden, and China. This demonstrated that the majority of collaborating countries have developed economies and technology industries.

**TABLE 1:** Top 10 countries and organizations on the research of insomnia in adolescents.

Rank	Countries	Counts	Organizations	Counts
1	The United States (North America)	898 (36.39%)	University of Pennsylvania (The United States)	60 (2.43%)
2	China (Asia)	303 (12.28%)	University of Pittsburgh (The United States)	38 (1.54%)
3	The United Kingdom (Europe)	235 (9.52%)	University of Bergen (Norway)	37 (1.50%)
4	Australia (Oceania)	161 (6.52%)	Harvard University (The United States)	34 (1.38%)
5	Canada (North America)	158 (6.40%)	Shandong University (China)	33 (1.34%)
6	Germany (Europe)	122 (4.94%)	The Chinese University of Hong Kong (China)	33 (1.34%)
7	France (Europe)	117 (4.74%)	University of Washington (The United States)	31 (1.26%)
8	Italy (Europe)	108 (4.38%)	The University of Hong Kong (China)	30 (1.22%)
9	Japan (Asia)	86 (3.48%)	The University of Melbourne (Australia)	30 (1.22%)
10	Sweden (Europe)	84 (3.40%)	University of Cincinnati (The United States)	30 (1.22%)

**FIGURE 3:**
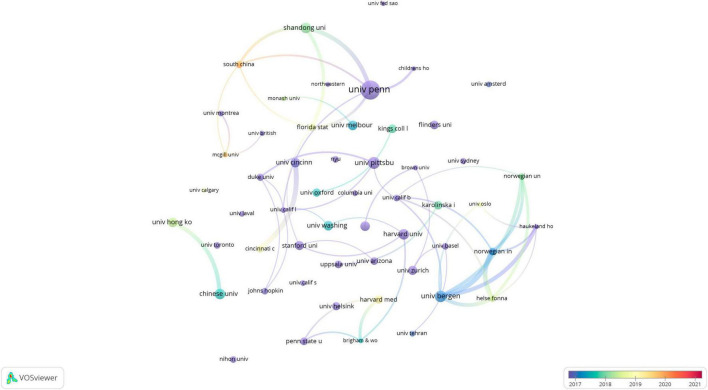
The visualization of organizations of adolescent insomnia research.

The top 10 universities were distributed over four countries: the United States, China, Norway, and Australia, with the United States accounting for half of them. The three universities that published the most papers were the University of Pennsylvania (the United States; *n* = 60,2.43%), the University of Pittsburgh (the United States; *n* = 38,1.54%), and the University of Bergen (Norway; *n* = 37,1.50%). With a minimum threshold of 15 published papers, 54 organizations were selected for visualization, and the collaborative network was constructed according to the number of published studies and the relationships between the published studies from each organization (Figure 3). As illustrated in Figure 3, the University of Pennsylvania collaborated closely with Shandong University (China), Florida State University (the United States), and South China Normal University (China); University of Pittsburgh collaborated strongly with Duke University (the United States), University of California, Los Angeles (the United States), and University of California, Berkeley (the United States); and the University of Bergen cooperated tightly with the Norway Public Research Institute, the Norwegian University of Science and Technology, and the Haukland Hospital (Norway). However, we noted that, despite the large volume of publications, the top three universities did not cooperate closely with each other.

### Active journals and co–cited journals

**FIGURE 4:**
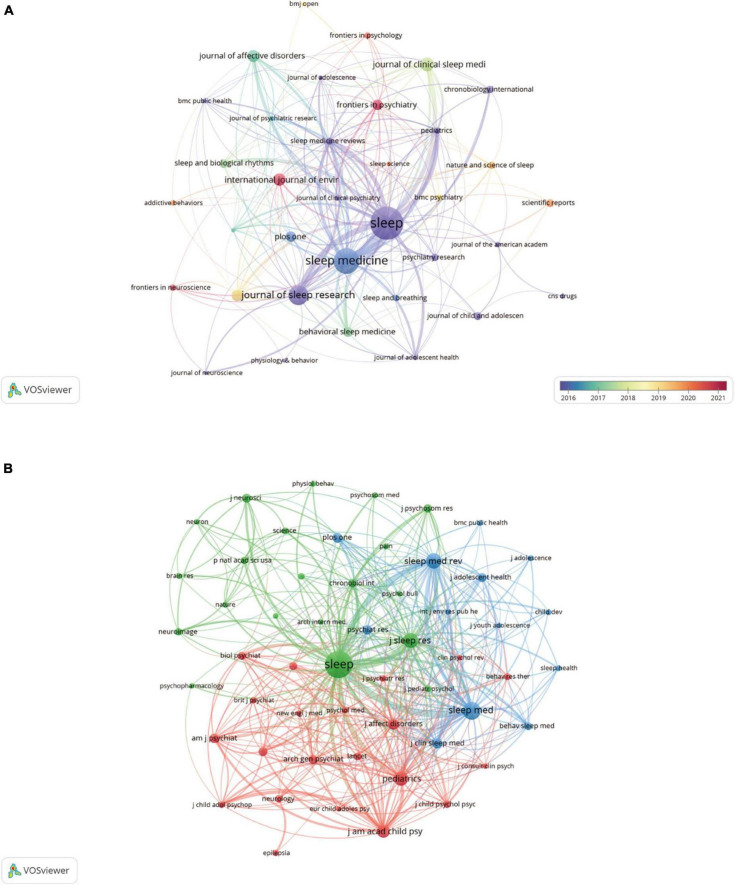
The visualization of journals **(A)** and co–cited journals **(B)** on research of insomnia in adolescents.

There were 935 publications published in journals on adolescent insomnia. Most papers were published in *SLEEP* (*n* = 137, 5.6%), followed by *SLEEP MEDICINE* (*n* = 100,4.1%) and *JOURNAL OF SLEEP RESEARCH* (*n* = 71, 2.9%) (Table 2). Among the top 10 journals, *JOURNAL OF AFFECTIVE DISORDERS* (impact factor [IF] = 6.533) had the highest impact factor, followed by *SLEEP* (IF = 6.313) and *FRONTIERS IN PSYCHOLOGY* (IF = 5.435) (Table 2). Citation relationships among journals such as *SLEEP, SLEEP MEDICINE*, *JOURNAL OF SLEEP RESEARCH*, and *JOURNAL OF AFFECTIVE DISORDERS* were quite active, as seen in Figure 4A. When viewed over time, the number and citation relationships of articles published in *SLEEP*, *SLEEP MEDICINE*, and *JOURNAL OF SLEEP RESEARCH* were primarily concentrated in an earlier period. While the number of articles published in *FRONTIERS IN PSYCHOLOGY*, *FRONTIERS IN PSYCHIATRY*, *FRONTIERS IN NEUROSCIENCE*, and *INTERNATIONAL JOURNAL OF ENVIRONMENTAL RESEARCH AND PUBLIC HEALTH* was less than that of *SLEEP*, the majority were published in recent years, and the citation relationship with other journals was primarily concentrated in recent years.

**TABLE 2:** Top 10 journals and co–cited journals for research of insomnia in adolescents.

Rank	Journals	Counts	IF	Q	Co–cited journals	Co–citations	IF	Q
1	Sleep	137 (5.6%)	6.313	Q1	Sleep	7351	6.313	Q1
2	Sleep Medicine	100 (4.1%)	4.842	Q2	Sleep Medicine	3091	4.842	Q2
3	Journal of Sleep Research	71 (2.9%)	5.296	Q2	Sleep Medicine Reviews	2314	11.401	Q1
4	Journal of Clinical Sleep Medicine	45 (1.8%)	4.324	Q2	Journal of Sleep Research	2184	5.296	Q2
5	International Journal of Environmental Research and Public Health	39 (1.6%)	4.614	Q2	Pediatrics	2076	9.703	Q1
6	Journal of Affective Disorders	37 (1.5%)	6.533	Q1	Journal of the American Academy of Child and Adolescent Psychiatry	1709	13.113	Q1
7	Sleep Health	35 (1.4%)	4.207	Q2	Journal of Affective Disorders	1162	6.533	Q1
8	Frontiers in Psychiatry	32 (1.3%)	5.435	Q2	Journal of Clinical Sleep Medicine	1099	4.324	Q2
9	PLoS One	32 (1.3%)	3.752	Q2	PLoS One	977	3.752	Q2
10	Behavioral Sleep Medicine	30 (1.2%)	3.492	Q3	American Journal of Psychiatry	899	19.248	Q1

Co–citation occurs when two journals are referenced in one or more identical papers at the same time. In this case, the two journals cited by the same third journal are thought to share intellectual affinity (43). Journal co–citation analysis may be used to identify the journals that are significant in a certain topic. As demonstrated in Table 2, among the top 10 co–cited journals, five journals were cited more than 2000 times, among which *SLEEP* (co–citations = 7351) was the most cited, followed by *SLEEP MEDICINE* (co–citations = 3091), *SLEEP MEDICINE REVIEWS* (co–citations = 2314), *JOURNAL OF SLEEP RESEARCH* (co–citations = 2184) and *PEDIATRICS* (co–citations = 2076). Additionally, the impact factor of *AMERICAN JOURNAL OF PSYCHIATRY* was the highest (IF = 19.248), followed by *JOURNAL OF THE AMERICAN ACADEMY OF CHILD AND ADOLESCENT PSYCHIATRY* (IF = 13.113). Fifty–four journals were chosen using a minimum co–citation threshold of 300 papers, and a co–citation relationship network map was created (Figure 4B). As shown in Figure 4B, *SLEEP* had a firm co–citation relationship with journals such as *SLEEP MEDICINE*, *SLEEP MEDICINE REVIEWS*, and *PEDIATRICS*.

**FIGURE 5:**
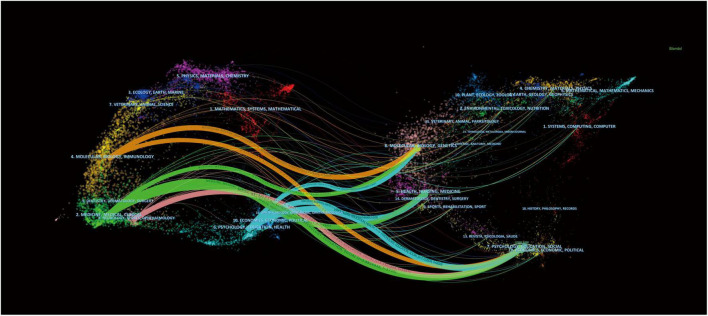
The dual–map overlay of journals and co–cited journals on the research of insomnia in adolescents.

Figure 5 was created using CiteSpace to visualize citation relationships between journals using a dual–map overlay, with the cited journal cluster on the left side and the co–cited journal cluster on the right. As illustrated in Figure 5, there were four citation paths. The orange path represents research published in molecular/biology/genetics journals, and psychology/education/social journals were mainly cited by literature in molecular/biology/immunology journals. The green path represents research published in molecular/biology/genetics journals, health/nursing/medicine journals, and psychology/education/social journals that was mainly cited by literature in medicine/medical/clinical journals. The pink path represents research published in psychology/education/social journals mainly cited by literature in neurology/sports/ophthalmology journals. The blue path represents research published in molecular/biology/genetics journals, health/nursing/medicine journals, and psychology/education/social journals that were mainly cited by literature in psychology/education/health journals. Research on adolescent insomnia has represented multiple research fields, including medical, health, social, psychology, and education.

### Active authors and co–cited authors

**FIGURE 6:**
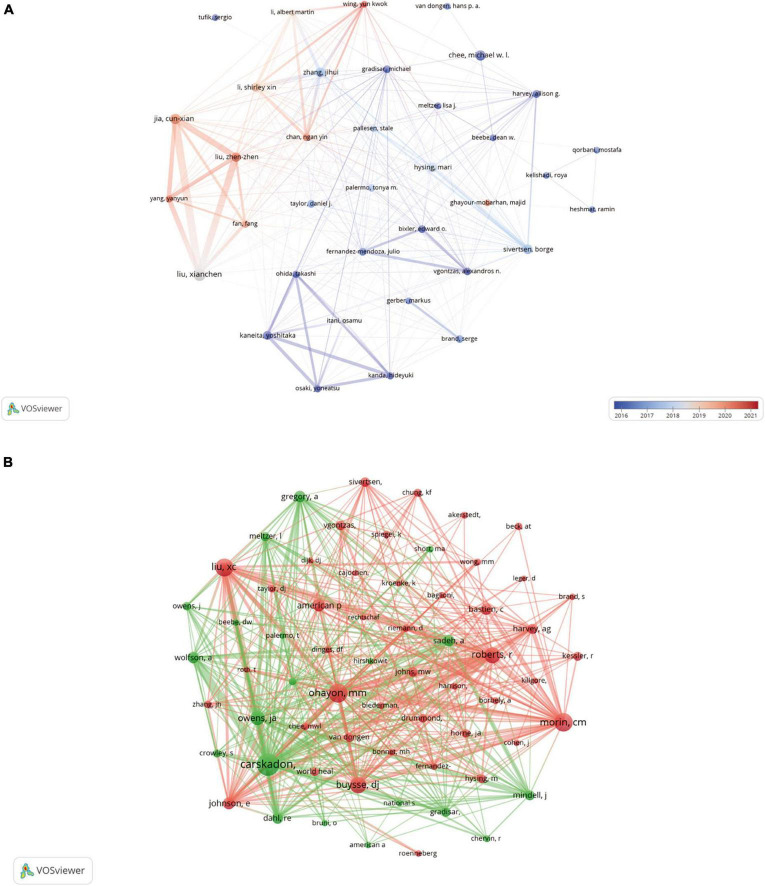
The visualization of authors **(A)** and co–cited authors **(B)** on research of insomnia in adolescents.

The types of literature included in this study were analyzed. A total of 10605 authors published studies on adolescent insomnia. Seven of the top 10 authors published more than 20 papers, while one author published more than 30 articles (Table 3). We analyzed the authors who published 10 or more articles, selected 36 authors, and drew an author network (Figure 6A). There was close cooperation between multiple authors. For example, Xianchen Liu had close cooperation with Cunxian Jia, Zhenzhen Liu, and other authors, and the cooperation time was most commonly nearly five years; Yoshitaka Kaneita had close cooperation with Takashi Ohida, Hideyuki Kanda, and other authors; Shirley Xin Li had active collaboration with Ngan Yin Chan, Yun Kwok Wing, and others. There were four authors who were co–cited more than 450 times among the 48305 co–cited authors: Mary A Carskadon (*n* = 669), Maurice M Ohayon (*n* = 530), Charles M Morin (*n* = 513), and Xianchen Liu (*n* = 487) (Table 3). These authors can be considered leaders in the field of adolescent insomnia research. Subsequently, we used a minimum threshold of at least 100 co–citations, and 64 authors satisfied the criterion; thus, we created a co–citation network diagram (Figure 6B). As seen in Figure 6B, there were positive collaborative relationships among different co–cited authors, such as Mary A Carskadon and Judith A Owens, Xianchen Liu; Maurice M Ohayon and Charles M Morin, Daniel J Buysse. There was a strong collaborative relationship between leading figures in this field of research.

**TABLE 3:** Top 10 authors and co–cited authors for research of insomnia in adolescents.

Rank	Authors	Counts	Co–cited authors	Citations
1	Xianchen Liu	34	Mary A Carskadon	669
2	Michael W L Chee	24	Maurice M Ohayon	530
3	Cunxian Jia	23	Charles M Morin	513
4	Mari Hysing	22	Xianchen Liu	487
5	Børge Sivertsen	22	Daniel J Buysse	436
6	Jihui Zhang	21	Robert E Roberts	421
7	Zhenzhen Liu	20	Judith A Owens	352
8	Yoshitaka Kaneita	18	Elizabeth O Johnson	277
9	Shirley Xin Li	17	Avi Sadeh	258
10	Ngan Yin Chan	14	Celyne H Bastien	256

### References analysis

**FIGURE 7:**
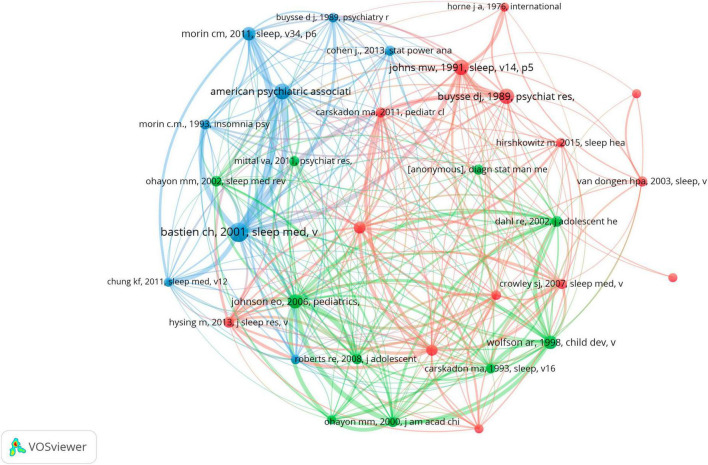
The visualization of co–cited references of adolescent insomnia research.

Over the past 21 years, 74,859 references on adolescent insomnia were co–cited. Co–cited reference means that a reference to be considered co–cited must have been co–cited in multiple publications and, therefore, can be considered as the basis of research in a particular field (42). Among the top 10 co–cited references (Table 4), the minimum number of co–cited was 92, and the highest number of co–citations was 234 times (44). We create a co–citation network based on references with 65 or more co–citations (Figure 7). As shown in Figure 7, “BASTIEN CH, 2001, SLEEP MED” (44) demonstrated robust co–citation relationships with “AMERICAN PSYCHIATRIC ASSOCIATION, 2013, DIAGNOSTIC STAT MANU” (1), “BUYSSE DJ, 1989, PSYCHIAT RES” (45) and “MORIN CM, 2011, SLEEP” (46).

**TABLE 4:** Top 10 co–cited references on the research of insomnia in adolescents.

Rank	Co–cited reference	Citations
1	Bastien CH, 2001, Sleep Med, V2, P297 (44)	234
2	American Psychiatric Association, 2013, Diagnostic Stat Manu, V5TH (1)	169
3	Buysse DJ, 1989, Psychiat Res, V28, P193 (45)	160
4	Johns MW, 1991, Sleep, V14, P540 (47)	158
5	Johnson EO, 2006, Pediatrics, V117, PE247 (12)	137
6	Wolfson AR, 1998, Child Dev, V69, P875 (48)	131
7	Morin CM, 2011, Sleep, V34, P601 (46)	129
8	Owens J, 2014, Pediatrics, V134, PE921 (49)	103
9	Gradisar M, 2011, Sleep Med, V12, P110 (50)	101
10	Ohayon MM, 2002, Sleep Med Rev, V6, P97 (51)	92

**FIGURE 8:**
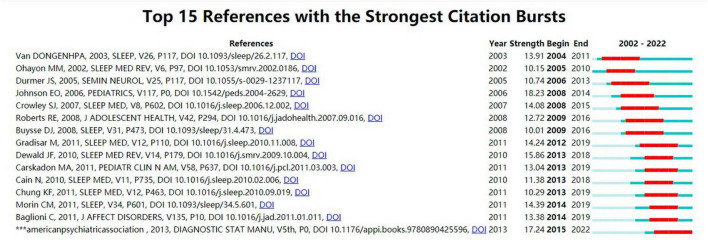
Top 15 references with strong citation bursts of adolescent insomnia research.

We used CiteSpace to analyze strong citation bursts and obtained 15 references. As illustrated in Figure 8, every bar indicates a year, and the red bar indicates the year of strong citation burstiness. References with citation bursts occurred as early as 2004 and as late as 2015. These 15 references had endurance strengths between 6 and 8 years, while their burst strengths varied from 10.1 to 18.23. The reference with the strongest citation burst (strength = 18.23) was titled “Epidemiology of DSM–IV Insomnia in Adolescence: Lifetime Prevalence, Chronicity, and an Emergent Gender Difference,” published in *PEDIATRICS*, authored by Eric O Johnson, et al.

The 15 references are included in Table 5 in the order of the literature in Figure 8, with the main research results summarized.

**TABLE 5:** The main research results of the 15 references with strong citations bursts.

Rank	Title	Authors	strength	Main research results
1	The cumulative cost of additional wakefulness: dose–response effects on neurobehavioral functions and sleep physiology from chronic sleep restriction and total sleep deprivation	Hans P A Van Dongen, Greg Maislin, Janet M Mullington, David F Dinges	13.91	Lack of sleep can seriously impair neurobehavioral function (52)
2	Epidemiology of insomnia: what we know and what we still need to learn	Maurice M Ohayon	10.15	Insomnia may harm adolescents’ learning, family, and social life, leading to depression (51)
3	Neurocognitive consequences of sleep deprivation	Jeffrey S Durmer, David F Dinges	10.74	Instability in the sleep–wake rhythm negatively affects mood, neurocognitive domains, cognitive performance, and motor function (53)
4	Epidemiology of DSM–IV insomnia in adolescence: lifetime prevalence, chronicity, and an emergent gender difference	Eric O Johnson,Thomas Roth,Lonni Schultz,Naomi Breslau	18.23	Girls are more likely to experience insomnia once menstruation begins due to pubertal development (12)
5	Sleep, circadian rhythms, and delayed phase in adolescence	Stephanie J Crowley, Christine Acebo, Mary A Carskadon	14.08	During puberty, developmental and environmental factors can cause changes in circadian mechanisms (13)
6	Chronic insomnia and its negative consequences for health and functioning of adolescents: a 12–month prospective study	Robert E Roberts, Catherine R Roberts, Hao T Duong	12.72	Chronic insomnia in adolescents has negative effects on interpersonal relationships and physical or psychological functioning (54)
7	Prevalence, course, and comorbidity of insomnia and depression in young adults	Daniel J Buysse, Jules Angst, Alex Gamma, Vladeta Ajdacic, Dominique Eich, Wulf Rössler	10.01	Insomnia was comorbid with, rather than secondary to, depression (55)
8	Recent worldwide sleep patterns and problems during adolescence: A review and meta–analysis of age, region, and sleep	Michael Gradisar, Greg Gardner, Hayley Dohnt	14.24	Adolescents had longer sleep delays and less total sleep as they aged, and the findings were more pronounced in Asian adolescents (50)
9	The influence of sleep quality, sleep duration and sleepiness on school performance in children and adolescents: A meta–analytic review	Julia F Dewald, Anne M Meijer, Frans J Oort, Gerard A Kerkhof, Susan M Bögels	15.86	The effects of insomnia on cognitive function and academic performance occur in early adolescence rather than late adolescence (56)
10	Sleep in adolescents: the perfect storm	Mary A Carskadon	13.04	With the increase of age and academic pressure, as well as the change in psychological state, the sleep delay time of adolescents will increase continuously (57).
11	Electronic media use and sleep in school–aged children and adolescents: A review	Neralie Cain, Michael Gradisar	11.38	Excessive use of electronic media in adolescents leads to sleep delays and reduced total sleep time (58)
12	Assessing insomnia in adolescents: comparison of insomnia severity index, Athens Insomnia Scale and Sleep Quality Index	Ka–Fai Chung, Katherine Ka–Ki Kan, Wing–Fai Yeung	10.29	The Chinese version of the Insomnia Severity Index (ISI) and Athens Insomnia Scale (AIS) have good psychometric characteristics, especially in the assessment and screening of insomnia symptoms in adolescents (59).
13	The insomnia severity index: psychometric indicators to detect insomnia cases and evaluate treatment response	Charles M Morin, Geneviève Belleville, Lynda Bélanger, Hans Ivers	14.39	Insomnia is strongly associated with psychological symptoms, fatigue, and poor health (60).
14	Insomnia as a predictor of depression: A meta–analytic evaluation of longitudinal epidemiological studies	Chiara Baglioni, Gemma Battagliese, Bernd Feige, Kai Spiegelhalder, Christoph Nissen, Ulrich Voderholzer, Caterina Lombardo, Dieter Riemann	13.38	Insomnia has a twofold risk of developing depression compared to people with no sleep difficulties (61)
15	Diagnostic and statistical manual of mental disorders	American Psychiatric Association	17.24	Detailed diagnostic criteria for insomnia and other mental disorders (1)

### Keywords analysis

**FIGURE 9:**
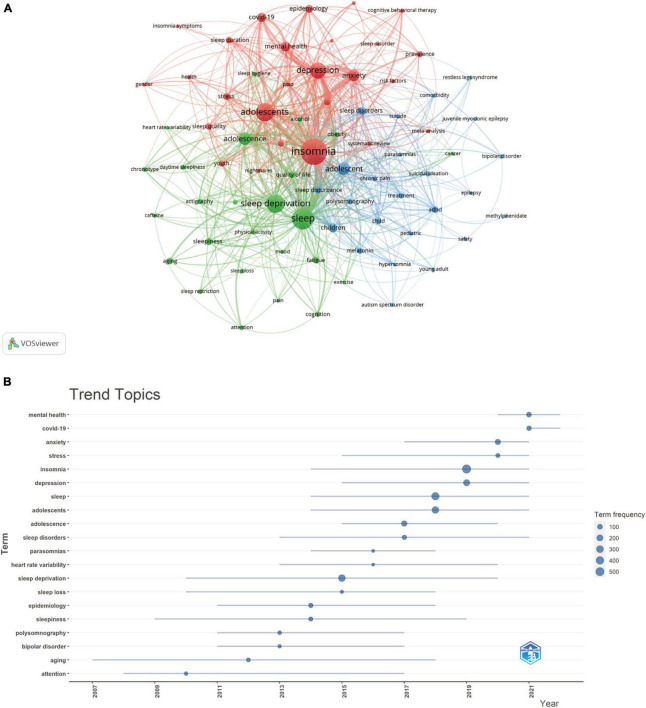
The visualization of frequency keywords **(A)** and trend topic analysis **(B)** on research of insomnia in adolescents.

By analyzing keywords, we can quickly recognize the research frontiers and hotspots in adolescent insomnia across time. The top 20 high–frequency keywords in studies on adolescent insomnia are included in Table 6. Among these keywords, depression was the most frequently used, except insomnia, sleep, sleep deprivation, and adolescents, which represented the main research direction of adolescent insomnia. Visual cluster analysis was performed using VOSViewer on keywords with a frequency of at least 15 (Figure 9A). The results were displayed in a network diagram; we obtained three clusters representing three research directions, and stronger connections between the keywords could be observed by analyzing the thickness of the lines connecting the nodes. Among the keywords in the red clusters were depression, anxiety, and mental health. Among the keywords in the blue clusters were chronic pain, attention–deficit hyperactivity disorder (ADHD), suicidal ideation, and melatonin. Among the keywords in the green clusters were fatigue, aging, obesity, and quality of life. The three clusters represented the mental health effects of insomnia (red), comorbidities of insomnia (blue), and behavioral effects of insomnia (green). Mental health, comorbidities and behavioral effects, thus, are the main research directions of adolescent insomnia.

**TABLE 6:** Top 20 keywords on the research of insomnia in adolescents.

Rank	Words	Counts	Rank	Words	Counts
1	Insomnia	547	11	Children	72
2	Sleep	375	12	Epidemiology	61
3	Sleep deprivation	283	13	Sleep disorders	58
4	Adolescents	279	14	Sleepiness	49
5	Depression	228	15	Stress	48
6	Adolescence	144	16	Quality of life	45
7	Adolescent	144	17	Sleep duration	43
8	Anxiety	128	18	ADHD	41
9	Mental health	91	19	Sleep disturbance	41
10	COVID–19	81	20	Sleep quality	41

Topic trend analysis was performed by R–bibliometrix, and Figure 9B was plotted according to the time distribution segments of each trending topic. Research on adolescent insomnia before 2014 focused on the physiology and pathology of insomnia, especially aging, bipolar disorder, and parasomnias. After 2014, more focus was placed on the influence of psychological factors on insomnia, especially in 2020–2022. The keywords “mental health” and “coronavirus disease 2019” (COVID–19) represented the current hotspots (a subject of study in a field for a period of time) and trends in adolescent insomnia research.

## Discussion

### General information

Among the articles included in this study, the diagnostic criteria selected were Diagnostic and Statistical Manual of Mental Disorders, Fourth Edition (DSM–IV) (62–64), Diagnostic and Statistical Manual of Mental Disorders, Fifth Edition (DSM–V) (65–67), and the International Classification of Sleep Disorders (ICSD) (68–70); the population was adolescents; and the disease was insomnia.

In period I (2002–2016), there were 1091 articles published, with an average of 73 publications published each year. According to Supplementary Figure 1, this phase was still in the stage of initial research in this field, but the research content increased. In period II (2017–2022), a total of 1377 publications were published during a six–year period, with an average of 230 papers published per year, which is about three times that of the period I. This indicated that research on adolescent insomnia was in an explosive growth period over these six years, and adolescent insomnia has attracted increased attention. This phenomenon may be related to a major report (Global Accelerated Action for the Health of Adolescents [AA–HA!]: Guidance to support country implementation) (71) published by the World Health Organization in May 2017. This has led researchers around the world to focus on adolescent health problems, such as insomnia.

The United States and China together accounted for about half of the world’s articles on adolescent insomnia and were the main countries studying adolescent insomnia worldwide; the United States (*n* = 898, 36.39%) produced three times as many articles as China (*n* = 303, 12.28%). Of the top 10 research institutions in this field, the majority are located in the United States (accounting for half of the total) and China (three institutions). There was strong and close cooperation among countries, mainly among the United States and China, Canada, the United Kingdom, and Australia. There was also close cooperation among various research institutions, mainly between the University of Pennsylvania and Shandong University, Florida State University, and South China Normal University; and between the University of Bergen and the Norway Public Research Institute, the Norwegian University of Science and Technology, and the Haukland Hospital. However, according to the analysis, even with the United States and China accounting for half of the world’s articles, close cooperation between countries was limited to a few economically and technologically developed countries. It means that the breadth and intensity of collaborative research between countries was not ideal and is not conducive to promoting and developing global research on adolescent insomnia. Similarly, research institutions in various countries also cooperated with a fixed number of universities rather than forming a global network of cooperative relationships, which is not beneficial to the long–term steady development of this field. As a result, we strongly recommend that research institutions from different countries develop more comprehensive and in–depth collaborative studies to boost global research networks on insomnia in adolescents.

An analysis of journals revealed that the majority of the research on adolescent insomnia was published in *SLEEP* (*n* = 137 [5.6%], IF = 6.313, Q1), which was the most common journal in the field, but it was not the journal with the highest impact factor. The journal with the highest impact factor among those publishing adolescent insomnia research was *JOURNAL OF AFFECTIVE DISORDERS* (*n* = 37 [1.5%], IF = 6.533, Q1). However, the difference in influence between these journals was small. Among the co–cited journals, *SLEEP* was the most cited and was the most influential journal overall. When combined with the timeline analysis, the main body of current adolescent insomnia research was in *FRONTIERS IN PSYCHOLOGY*. This showed that adolescent insomnia is receiving increased attention in more journals. The majority of the co–cited journals were Q1 and Q2 journals that provide high–quality literature support for the study of insomnia in adolescents. It is worth noting that current research on adolescent insomnia is mostly published in clinical research journals, such as those related to insomnia, psychology, and behavior, and few studies have been published in more general basic research journals, and the pathological mechanisms of adolescent insomnia remain unclear.

According to the analysis of authors, Xianchen Liu (*n* = 34) ranked first in the number of articles published. Most of the articles written by Xianchen Liu indicated that stressful life events, non–suicidal self–injury, and suicidality were partially mediated by insomnia and frequent nightmares (72–74). There are several clinical diseases and symptoms with increased risk of comorbidity with insomnia, such as ADHD, restless legs syndrome, and frequent pain (75–77), and menstrual pain and irregular menstruation appear to be linked to insomnia (78). Sleep deprivation may also contribute to stress in general life and poor academic performance, which can increase the risk of depression (79, 80). Overall, the studies by Xianchen Liu explained and analyzed the predisposing factors, comorbidity factors, and effects of insomnia in adolescents, but the problem is that the vast majority of these studies involved Chinese adolescents, and they cannot fully represent the current situation of insomnia in adolescents worldwide. This means that global multi–center collaborative research is required to better investigate the pathophysiology and risk factors of adolescent insomnia as well as its complications.

Analysis from the perspective of co–cited authors, Mary A Carskadon (citations = 669) was the most frequently cited author, followed by Maurice M Ohayon (citations = 530), Charles M Morin (citations = 513), and Xianchen Liu (citations = 487). In 2004, Mary A Carskadon wrote that the biological processes that regulate the body’s sleep/wake system mature during human development, a physiological phenomenon that is most likely caused by the amount and duration of sleep during adolescence (81). This idea established the basis for the theory that puberty affects sleep–wake rhythms. Subsequently, in 2009, a review published by Mary A Carskadon summarized that several components of the circadian system change throughout puberty, including the free–running period, continuous and discrete entrainment mechanisms, and recovery from photic phase shifts (82). The next year, Mary A Carskadon proposed that asymmetrical frequency–specific reductions in sleep electroencephalogram spectral power with early adolescent development may indicate early symptoms of cortical synaptic pruning in healthy adolescents (83). Accordingly, the research focus in this period mainly revolved around the physiological and pathological factors and pathogenesis of insomnia. However, a study published in 2013 showed that not only biological factors but also many external factors, such as societal (school–start times), familial (parent–set bedtimes), and personal factors (extra–curricular load), affect adolescent sleep (84). An experimental study in 2017 found that insomnia negatively affected sustained attention in adolescents (85). In the same year, a review published by Mary A Carskadon showed that the number of depressive symptoms was negatively correlated with the length of sleep and positively correlated with sleep latency and the number of awakenings (86). Adolescents who sleep for fewer than 5 hours each night are more likely to experience depression, anger management difficulties, confusion, and less energy and happiness compared with typical adolescents; with the emergence of insomnia, the incidence of emotional disorders and dysregulation in adolescents is rising (87). Thus, in recent years, Mary A Carskadon’s research focus has shifted from the pathogenesis of insomnia to the relationship between the negative effects of insomnia and comorbidities. Mary A Carskadon, one of the academic leaders in adolescent insomnia, highlighted that the main direction of current adolescent insomnia research focuses on the adverse effects and comorbidities of insomnia.

We initially conducted a co–citation analysis of references. We selected the top 10 articles with the most co–citations for bibliometric analysis to clarify the basis of research on adolescent insomnia. Bastien Celyne et al. published the most co–cited study in 2001, and this study examined the internal consistency and concurrent validity of the Insomnia Severity Index (ISI) in patients with insomnia. The results confirmed that the ISI was a reliable and valid method to quantify the severity of insomnia in young patients, as well as in patients with primary and secondary insomnia, which could be used to guide clinicians in deciding whether the presentation of insomnia meets clinical/diagnostic thresholds, and can also be used to evaluate treatment outcomes (44). At the same time, this study laid a foundation for the evaluation of the severity of insomnia and provided a basis for the diagnosis and treatment evaluation of adolescent insomnia. *Diagnostic and Statistical Manual of Mental Disorders: DSM–V* ranked second in the top ten 10 total co–cited references; this manual, developed by the American Psychiatric Association, details diagnostic criteria for insomnia and other psychiatric disorders (1). This was followed by the Pittsburgh Sleep Quality Index, which is a commonly used clinical sleep scale (45). The Epworth Sleepiness Scale is a trial–validated scale for evaluating daytime sleepiness (47). The ISI is a reliable and accurate tool for identifying insomnia in the general population and is sensitive to patient responses to therapy (46). An epidemiological study of adolescent insomnia based on the DSM–IV found that insomnia in adolescents was often chronic and showed sex differences, which may have been related to menstruation (12). Sleep–deprived adolescents experience daytime sleepiness, depressed mood, and sleep/wake behavioral difficulties (48). One review described the causes and consequences of insomnia in adolescents, including the presence of puberty, electronic media use, caffeine use, pain, chronic diseases, and mental health problems. Further, sleep deprivation was shown to increase the risk of depression, mood disorders, suicidal ideation, and obesity (49). Globally, adolescents experience longer sleep delays and less total sleep as they age, a phenomenon that is influenced by geography and culture, and is more pronounced among Asian adolescents. Asian adolescents sleep less than European adolescents and go to bed later than North American adolescents. The differences in cultural factors among different regions can be seen in school night bedtimes, school sleep habits, school night total sleep time, coursework, social practices, and other activities (50). Other cultural factors, such as school start times, extra–curricular activities, parental bedtime rules, and paid work, also play a role in adolescent insomnia. For example, in southern Australia, high school start times range from roughly 8:20 – 9:00 in the morning, compared with roughly 7:45 in the United States, and 5% of parents in the United States and 17% of parents in Australia set their adolescent’s bedtimes (84). As can be seen, the cultural differences between these regions may result in differences in insomnia symptoms (delayed sleep, insufficient total sleep time, or daytime fatigue or sleepiness) among adolescents in different regions. An epidemiological review of insomnia summarized the current status of insomnia research and put forward relevant recommendations. Major depressive disorders are more common among people who have insomnia, and ongoing insomnia is linked to the onset of new depressive episodes. Future epidemiological research should concentrate on the natural evolution of insomnia; the epidemiological genetic linkages of insomnia should also be examined, and more attention should be placed on differentiating between the many subtypes of insomnia (51). In conclusion, the top 10 co–cited references focused mainly on the following topics: developments and applications of insomnia measurement scales, risk factors for insomnia in teenagers, and emotional and behavioral issues brought on by sleeplessness. These represent the basis and directions of clinical research on adolescent insomnia. This also demonstrated what scholars and medical students who are new to the field need to know most.

References with bursts in citations highlighted the most important current subjects in this field of study, as they have been widely used as references in recent years. According to the analysis of the main contents of the references with citation bursts (Table 5), the following aspects were the main directions of research on adolescent insomnia: physiological factors, social factors, cultural factors, and environmental factors; the negative effects of insomnia on individual emotional, cognitive, behavioral, and motor function in adolescents; comorbidities of insomnia; and diagnosis, severity assessment tools, and treatment of insomnia in adolescents. According to the dual–map overlay analysis, numerous research fields were represented in the study of adolescent insomnia. It means that the incidence of insomnia in adolescents is not caused by a single factor, and the negative effects and comorbidities associated with it are similarly varied. As a result, many research fields must collaborate to create interdisciplinary interaction and jointly enhance research advancement.

### Hot spots and trends

The analysis of keywords and topic trends can help to swiftly explain details on research directions in a given field. Analysis based on high–frequency keywords and topic trends showed that, in addition to insomnia, sleep, sleep deprivation, adolescents, and sleep disorders, the following keywords were dominant: depression, anxiety, mental health, COVID–19, stress, quality of life, heart rate variability, attention, and ADHD. As shown in Figure 9A, there were three clusters of keywords, representing mental health (red), comorbidities (blue) and behavioral effects (green), which represented the main aspects of adolescent insomnia research. Comorbidities and behavioral effects can be summarized as “disease,” and “mental health” can be a separate aspect of research. Therefore, the current trends in adolescent insomnia research include the following aspects.

### Mental health

From the results of the bibliometric analysis (Table 6), depression (*n* = 228), anxiety (*n* = 128), mental health (*n* = 91), and stress (*n* = 48) were frequently appearing keywords. Depression and stress were trend topic from 2014–2021; anxiety was a trend topic from 2016–2021, and mental health was from 2020–2022 (Figure 9B). It is possible that since 2014, mental disorders have progressively become a focus of adolescent insomnia researchers, although, at this moment, studies have focused on single diseases such as depression/stress/anxiety, not mental health in general until 2020. Starting in 2020, the focus of many researchers has shifted from single comorbidities to mental health, which may be related to insomnia and multiple mental diseases comorbidities caused by COVID–19 (88, 89). This means that in recent years, the trend topics have changed from a single psychological comorbidity to the relationship between insomnia and overall mental health.

Depression, anxiety, and stress as keywords represent psychological disorders and can be summarized as “mental health.” Based on diagnostic interview data from the Adolescent Supplement to the National Comorbidity Survey, it was estimated that 49.5% of adolescents (13–18 years) experience mental disorders (90). Adolescent insomnia is closely related to adolescent mental health, and poor mental health (especially depression and anxiety) and stress from school and society are important risk factors for insomnia (16, 91, 92). Insomnia and mental health problems commonly first arise in puberty, and girls are more likely than boys to experience both (93). Poor long–term mental health can develop into mental disorders. Moreover, the comorbidity of insomnia and mental disorders can further enhance the effects of insomnia symptoms on physical and mental health. The incidence of negative behaviors (especially suicidal ideation) in adolescents with comorbid insomnia and mental disorders is higher than that in adolescents with mental disorders alone (94). Depression and anxiety, as risk factors for insomnia, can easily induce insomnia (6). Adolescents face tremendous stress during puberty, including academic pressure, social pressure, and parental and social expectations. Similarly, daily stress negatively affects sleep, and there is a reciprocal link between daily stress and sleep (15). The symptoms bridging insomnia, depression, anxiety, and stress are trouble relaxing and low energy (95). Insomnia interacts with mental health disorders, including depression and anxiety, with insomnia being most strongly associated with depression (95). Insomnia can increase the risk of depression, Liu et al. found (96) that insomnia in adolescents could increase the risk of depression 5 –9 times. Adolescents with depression slept on average 1 –1.5 hours less than adolescents without depression, while sleep onset latency and waking after sleep onset over 2 hours increased their risk of depression by six–fold; 5 –6 hours of sleep increased their risk of depression three–fold; and sleeping less than 5 hours increased their risk of depression 6 –9 times (97). Mental disorders and sleep disorders are physiologically connected, often by dysfunction of the hypothalamic adrenal pituitary axis. Hypothalamic hyperactivity in patients with major depression is associated with sleep fragmentation and shortened sleep times, and elevated cortisol secretion is associated with reduced rapid eye movement latency in adolescent depression (98). Depression may result from insomnia because it interferes with the processing of rewards by changing activity in prefrontal cortex regions involved in emotional regulation (99). Insomnia in early puberty may induce depressive symptoms and affect mental health and cognitive–behavioral functioning by altering corticolimbic circuits through excessive arousal and sleep deprivation (99). Another academic view is that arousal cues in bed, excessive arousal, and delayed sleep are all potential causes of and contributors to depression (100). Cognitive behavioral therapy (CBT) is the first–line treatment for comorbid insomnia and mental health disorders, and the clinical use rate accounts for more than half of all clinical treatment methods (101, 102). By treating cognitive symptoms and deficits, enhancing stress management, and improving sleep architecture to reduce limbic activation, improve prefrontal regulation, and reduce mid–limbic brain dysregulation as well as better cortisol reactions to stress and inflammatory cytokine regulation, CBT reduces the symptoms of insomnia and comorbid mental health disorders, including anxiety and depression (103). Therefore, CBT can effectively improve depression and anxiety in adolescents with adolescent insomnia through mental health education, cognitive restructuring, and mental and physical relaxation techniques (104–106).

### Disease

In the keyword analysis (Table 6), “COVID–19” (*n* = 81) and “ADHD” (*n* = 128) appeared frequently. The topic trend analysis (Figure 9B) shows that “COVID–19” has become a topic trend since 2021, and “heart rate variability” has continued to be a topic trend starting in 2013. The reason why “COVID–19” has become a topic trend may be related to the global pandemic, but as it has been gradually controlled, it will likely cease to be a topic trend in the future. At this stage, it is still possible to study trends among “heart rate variability” and “ADHD.”

It is imperative to acknowledge that the causes and consequences of insomnia among adolescents are frequently interwoven in a convoluted and intimate manner, and the reciprocal effects of these factors exacerbate this, culminating in a deleterious cycle. There are additional comorbidities associated with insomnia, including the aforementioned depression, and some of the symptoms caused by comorbid insomnia contribute to declines in quality of life, impeding the development and maturation of adolescents (107). During the COVID–19 pandemic, physical and mental health was negatively affected in adolescents, leading to sleep delays, insomnia, depression, decreased learning ability, cognitive impairment, and an increased risk of comorbidities (108, 109). Bothe et al. (110) found that adolescents affected by the epidemic reported sleep disturbances, diminished sleep quality, nightmares, anxiety, and depression, and as a result of these symptoms, daytime fatigue, impaired academic and social functions, and a diminished capacity to regulate emotions and emotional responses. This study also showed that, during the pandemic, the number of individuals with insomnia nearly doubled in comparison to prior years, and anxiety associated with COVID–19 and the incidence of sleep disturbances were positively related, particularly among younger adolescents (110).

Several aspects of health, such as blood pressure and cardiac homeostasis, are directly affected by sleep (111). Irregular sleep patterns (such as intermittent insomnia) in adolescents have adverse effects on cardiac autonomic regulation and may cause fluctuations in heart rate (112). A study in adolescents showed a negative correlation between heart rate variability and sleep–related problems (113). The role of insomnia in the development of heart rate variability should be considered in the study and treatment of heart rate variability in adolescents where the determination of the cause is difficult.

ADHD combined with sleep disorders can seriously interfere with learning in adolescents and the quality of life in families (114). ADHD is characterized by delayed neuromodulation, neurocognitive deficits, and psychosocial stress, which interact to cause physical, psychological, and behavioral dysfunction. Insomnia is also associated with these risk factors and may exacerbate ADHD (115). Adolescents with ADHD are more likely than their peers to experience sleep disturbances and have altered circadian function (116); they can also experience inattention and oppositional emotions due to insomnia (117). Symptoms of ADHD and sleep disturbances share neural correlates, such as structural alterations in the ventral attention system and front striatal circuitry, and ADHD symptoms significantly mediate the association between these structural brain abnormalities and insomnia. Genes involved in neurotransmission and circadian entrainment are also differentially expressed in the involved brain regions (118). In a study by Becker et al. (119), insomnia, as measured by sleep monitoring, daily affect, emotional regulation, and internalizing symptoms, was found to be the cause of common mood and emotional disorders in adolescents with ADHD. Adolescents with ADHD had elevated levels of perceived stress and sleep problems, and co–occurring insomnia and ADHD symptoms moderate the effects of inattention and hyperactivity (120). When conducting in–depth studies of comorbid ADHD and insomnia, it is important to consider the interactions between somatic, psychological, stress, and behavioral dysfunctions, and comorbid symptoms are potential intervention targets that may improve treatment responses.

### Advantages and shortcomings

This bibliometrics analysis provided specific advantages. On the one hand, there were not any published studies on adolescent insomnia using bibliometrics. This study employed a bibliometric approach to examine the present research environment around multimodal imaging tools. We used three types of bibliometrics software to perform bibliometric and visual analysis and analyzed publications on adolescent insomnia over a 21–year. The entire analysis process was objective, and objective results were obtained. On the other hand, this systematic analysis of adolescent insomnia research provides a comprehensive guide for scholars concerned with this research field, as well as a more objective and comprehensive hot spot and trend analysis than traditional reviews, which also forecast future research trends.

Unfortunately, there were some shortcomings in the present study. First, the data in this study were only obtained from the WoSCC database, and other databases were neglected. Therefore, some relevant studies may have been overlooked. Second, this study focused on the annual number of publications, countries, organizations, journals, authors, references, and keywords in this field; hence, some essential details were not included. Third, we only considered studies published in English, which might have introduced bias against publications written in other languages. Fourth, the search strategy was limited to the period from 1 January 2002 to 31 December 2022; this study did not include articles published after this date. Finally, bibliometric analysis methods cannot yet analyze complete texts of publications, and some information may have been missed, which can potentially be a disadvantage. To compensate for these shortcomings and defects, this study introduced the main patterns and trends of research based on highly cited publications and references with strong citation bursts and summarized the hot topics and trends based on keyword analysis.

## Conclusion

The number of articles on adolescent insomnia published each year has been increasing annually, indicating that adolescent insomnia has attracted more and more attention globally, and more scholars and clinicians should participate in research on adolescent insomnia. The top two nations in this field were the United States and China; however, their cooperative partners were limited to economically and technologically advanced countries. To increase the quality of global cooperative research, there is a need to strengthen the variety of collaboration between nations and institutions. As academic leaders in adolescent insomnia, research from Mary A Carskadon and Xianchen Liu reflects the current research emphases on adolescent insomnia and serves as a reference for clinicians, academics, and medical students. *SLEEP* has been one of the most influential journals that researchers may read to keep track of hot topics and the latest research. The references with the most co–citations represent the most beneficial and influential in the field and are the most valuable for medical students to study. The two main directions of current research on adolescent insomnia are mental health and disease, which necessitates a strengthening of research on the inducing factors of adolescent insomnia, particularly the mechanisms behind psychological factors and comorbidities in the occurrence and development of adolescent insomnia. This will provide a more favorable basis and new direction for the diagnosis and accurate treatment of adolescent insomnia. We should further increase research into the occurrence and development of diseases, psychological disorders, and behavioral disorders caused by insomnia, reduce the incidence of insomnia and behavioral disorders caused by insomnia, and provide more accurate treatment recommendations for adolescents with insomnia. In addition, research trends in mental health, comorbidities, and quality of life should be further explored. It is worth emphasizing that while developing basic research, clinical research on adolescent insomnia, including accurate insomnia assessment, insomnia diagnosis and safe and effective treatment methods, should be strengthened to complete the translation of research results into clinical services.

In summary, this study obtained new knowledge through bibliometric and visual analyses, such as the annual publication circulation (after 2017, it entered a period of significant growth) and major countries (The United States and China), institutions (University of Pennsylvania), and academic leaders (Xianchen Liu and Mary A Carskadon), the most influential journals (*SLEEP*), and the most influential research articles in research in the field of adolescent insomnia. It further provided a summary of two main focuses (mental health and disease) of current research and aimed to predict future trends in adolescent insomnia research. This new information may inform scholars, clinicians and medical students of the most influential institutions, authors, and literature and the focus of past research, the main aspects of current research, and predictions of future research trends.

## Data availability statement

The raw data supporting the conclusions of this article will be made available by the authors, without undue reservation.

## Author contributions

TG and YT wrote the manuscript. QW, JL, ZD, and YX analyzed the data and drew figures. FC and JM reviewed and revised the manuscript. All authors read and approved the final manuscript, contributed to the article, and approved the submitted version.

## Footnotes

1
https://www.bibliometrix.org/home/

## Supplementary material

Supplementary Figure 1 Annual publications of adolescent insomnia research.
